# Public Awareness of the Fencing Response as an Indicator of Traumatic Brain Injury: Quantitative Study of Twitter and Wikipedia Data

**DOI:** 10.2196/39061

**Published:** 2023-03-17

**Authors:** Kyle L Roe, Katherine R Giordano, Gary A Ezzell, Jonathan Lifshitz

**Affiliations:** 1 Department of Psychiatry, University of Arizona College of Medicine – Phoenix Phoenix, AZ United States; 2 Phoenix Veteran Affairs Health Care System Phoenix, AZ United States

**Keywords:** athlete, brain, concussion, fencing response, health communication, health information, injury pattern, posture, public education, science communication, social media, sport, trauma, traumatic brain injury

## Abstract

**Background:**

Traumatic brain injury (TBI) is a disruption in normal brain function caused by an impact of external forces on the head. TBI affects millions of individuals per year, many potentially experiencing chronic symptoms and long-term disability, creating a public health crisis and an economic burden on society. The public discourse around sport-related TBIs has increased in recent decades; however, recognition of a possible TBI remains a challenge. The fencing response is an immediate posturing of the limbs, which can occur in individuals who sustain a TBI and can be used as an overt indicator of TBI. Typically, an individual demonstrating the fencing response exhibits extension in 1 arm and flexion in the contralateral arm immediately upon impact to the head; variations of forearm posturing among each limb have been observed. The tonic posturing is retained for several seconds, sufficient for observation and recognition of a TBI. Since the publication of the original peer-reviewed article on the fencing response, there have been efforts to raise awareness of the fencing response as a visible sign of TBI through publicly available web-based platforms, such as Twitter and Wikipedia.

**Objective:**

We aimed to quantify trends that demonstrate levels of public discussion and awareness of the fencing response over time using data from Twitter and Wikipedia.

**Methods:**

Raw Twitter data from January 1, 2010, to December 31, 2019, were accessed using the RStudio package academictwitteR and queried for the text “fencing response.” Data for page views of the Fencing Response Wikipedia article from January 1, 2010, to December 31, 2019, were accessed using the RStudio packages wikipediatrend and pageviews. Data were clustered by weekday, month, half-year (to represent the American football season vs off-season), and year to identify trends over time. Seasonal regression analysis was used to analyze the relationship between the number of fencing response tweets and page views and month of the year.

**Results:**

Twitter mentions of the fencing response and Wikipedia page views increased overall from 2010 to 2019, with hundreds of tweets and hundreds of thousands of Wikipedia page views per year. Twitter mentions peaked during the American football season, especially on and following game days. Wikipedia page views did not demonstrate a clear weekday or seasonal pattern, but instead had multiple peaks across various months and years, with January having more page views than May.

**Conclusions:**

Here, we demonstrated increased awareness of the fencing response over time using public data from Twitter and Wikipedia. Effective scientific communication through free public platforms can help spread awareness of clinical indicators of TBI, such as the fencing response. Greater awareness of the fencing response as a “red-flag” sign of TBI among coaches, athletic trainers, and sports organizations can help with medical care and return-to-play decisions.

## Introduction

### Traumatic Brain Injury

Traumatic brain injury (TBI) is broadly defined as a disruption in normal brain function caused by an impact of external forces on the head or skull. TBI is both a public health crisis and an economic burden. In 2013, there were approximately 2.5 million TBI-related emergency department (ED) visits, approximately 282,000 TBI-related hospitalizations, and approximately 56,000 TBI-related deaths in the United States [1]. However, these numbers are likely to be underestimated as many individuals do not seek medical attention after a TBI. In the United States, children (aged 0-4 years), adolescents (aged 15-19 years), and older adults (aged >75 years) have the highest rates of TBI-related ED visits and hospitalizations [2]. Accurate on-site detection of TBI can guide patients to health care facilities. The prevalence of long-term disability associated with TBI has been estimated at 1.1% (3.17 million people) among the civilian population of the United States [3]. Nationwide, TBI is estimated to be responsible for approximately US $48 billion to US $76 billion in total costs, consisting mostly of indirect costs from lost productivity and absenteeism [4]. Early and accurate detection may reduce the health care and economic burden of TBI.

The most common types of TBI occur after rotational or acceleration/deceleration forces displace the brain within the skull without penetrating the skull (diffuse TBI) [5,6]. The pathophysiological damage can be widespread and may vary by severity of the impact forces (ie, mild, moderate, and severe), resulting in an array of persistent clinical symptoms. Symptoms can be broadly categorized as cognitive, somatic, sensory, motor, and emotional dysfunction, and each individual who sustains a TBI will likely experience a distinct range of clinical symptoms, symptom onset, and symptom resolution [2]. However, for most individuals who sustain a TBI, clinical symptoms resolve within 1-10 days after injury [7]. Persistent symptoms may linger for some individuals, particularly those who sustain repeated TBIs, for months to years after the injury, including increased risk for psychiatric disorders, drug abuse, and suicide [2,8,9].

Perhaps due to the potential for long-term symptoms and disability, considerable public interest focuses on sport-related TBI among adolescents (<19 years) [10,11]. The most common sport and recreational activities associated with TBI-related ED visits in adolescents are bicycling, American football, basketball, soccer, and playground activities [2]. From 2001 to 2009, the rate of adolescent ED visits related to a TBI associated with sports or recreational activities increased 62% [2]. While the rate of TBI-related ED visits increased, the rate of TBI-related hospitalizations and death remained stable over the same time. One reason for these trends could be increased awareness of TBI through public health campaigns, education, and sports media coverage, which could encourage individuals to seek medical treatment.

Recognition of a potential TBI and removing an athlete from play are the first steps to provide necessary medical care in the event of a TBI. However, mild to moderate diffuse TBI may not immediately present with overt clinical symptoms, putting individuals at risk for additional injuries if not removed from play. Current diagnostic tools for TBI vary in their accuracy, and recognition of a potential injury remains a challenge, prompting the need for additional screening criteria at or near the time of injury [12,13].

### Fencing Response

The fencing response is an immediate tonic posturing of the limbs, similar to the asymmetric tonic neck reflex in infants, where extension and flexion of opposite arms occur despite body position or gravity. Upon impact to the head, an individual demonstrating the fencing response immediately exhibits extension in 1 arm and flexion in the contralateral arm while falling to the ground; different manifestations have been observed between limbs. Tonic posturing is then retained for several seconds and readily observable by onlookers (eg, coaches, trainers, parents, and teammates). In 2009, Hosseini and Lifshitz [14] published their experimental and clinical research on the relationship between the fencing response and TBI. In the review of videos of people (including athletes) subjected to a head impact, they found that 66% of the participants demonstrated the fencing response upon impact, regardless of the side of the impact [14]. The average duration of tonic arm posturing was approximately 6 seconds.

The fencing response was additionally observed in adult male rats subjected to brain injury forces delivered by midline fluid percussion. However, the fencing response was observed only with brain injury forces of moderate magnitude and not mild magnitude or in uninjured sham animals [14]. Histopathological analysis of the lateral vestibular nucleus—the anatomical structure involved in the asymmetric tonic neck reflex in infants—further demonstrated transient neurochemical activation with injury [14]. The proximity of the lateral vestibular nucleus to the cerebellar peduncles makes it vulnerable to rotational mechanical forces that cause neuronal depolarization and elicit a neuromotor response, distinct from convulsion, associated with TBI.

The fencing response can thus be used as a visible indicator of TBI and may assist with screening and decisions regarding medical care [15-17]. In order to reach a broad audience, our research team created a Wikipedia page on the subject shortly after the publication. Wikipedia is widely used as a resource for medical information as it is easily accessible on the internet [18-20]. By accessing the Fencing Response Wikipedia page, health care professionals and the general public could educate themselves about the fencing response and link to the primary publication for more information [18,21,22]. Wikipedia pages can be updated as new information about the fencing response is discovered [20,23]. Additionally, the research team was active on Twitter shortly after the publication to demonstrate occurrences of the fencing response in professional sports and share links to both the Wikipedia page and original scientific publication. Twitter has hundreds of millions of daily users and has been shown to be an effective platform for education and spreading public health awareness [24,25]. The objective of this study was to quantify trends over time of the awareness of the fencing response using data extracted from Twitter and Wikipedia.

## Methods

### Twitter

First, we applied for academic research access for Twitter’s Academic Research Product Track v2 application programming interfaces (APIs) [26]. Once approved, raw data from Twitter were accessed using the RStudio package academictwitteR [27]. Approved academic research access was required to query the full Twitter archive. academictwitteR was designed for querying the Twitter Academic Research Product Track v2 API and was chosen for straightforward data collection and management. Tweets were queried for the text *fencing response* between January 1, 2010, and December 31, 2019. Between 2010 and 2019, a total of 6176 tweets contained the words *fencing* and *response*. These included both primary tweets and retweets, but not “liked” tweets. All tweets were read and verified for relevance to TBI. Unverified tweets contained the words *fencing* and *response*, but not in reference to TBI. Excluded tweets referenced, for example, the USA Fencing team’s response to misconduct allegations. A total of 2550 tweets were excluded, and 3626 tweets were included in the analysis. The number of tweets was analyzed as a function of time, including the day of the week, month, 6 months (September to February and March to August to represent the American football season vs off-season), and year. We performed a seasonal regression analysis in Microsoft Excel to further examine the relationship between the number of fencing response tweets and month of the year. After data were visualized, we identified peaks at certain months and years (ie, December 2017). A rereview of the Twitter raw data during the peaks was used to identify notable events that initiated the discussion on Twitter.

### Wikipedia

Raw data from Wikipedia were accessed using the RStudio packages *wikipediatrend* and *pageviews* [28,29]. Wikipediatrend and pageviews were developed in tandem to easily access daily page view data from “Wikimedia” sites and were chosen for feasibility. Daily page views for the *Fencing Response* Wikipedia article [30] were accessed between January 1, 2010, and December 31, 2019, for analysis. The number of page views was analyzed as a function of time, including day of the week, month, 6 months (September to February and March to August to represent the American football season vs off-season), and year. We performed a seasonal regression analysis in Microsoft Excel to further examine the relationship between Wikipedia page views and month of the year, using the month of May as a reference. Wikipedia page views on specific days around the notable events identified by Twitter are reported.

### Ethical Considerations

Prior to the start of the study, we submitted a project proposal to the University of Arizona’s institutional review board. The institutional review board determined that the study (STUDY 00000842) did not involve human subjects research as defined by Department of Health and Human Services and Food and Drug Administration regulations. Public Twitter data (ie, tweets from nonprivate Twitter accounts) posted over the last 7 days are available to any user with a Twitter account that registers on the Twitter Developer Platform. Further, a developer account can apply to access the academic research product track to gain access to the full archive of tweets dating back to March 2006. With academic approval from Twitter for the current research project, an authentication key provided unrestricted access to the full archive of tweets.

## Results

### Twitter Mentions of the Fencing Response Increased Over Time and Have Patterns of Seasonality

**Figure 1:**
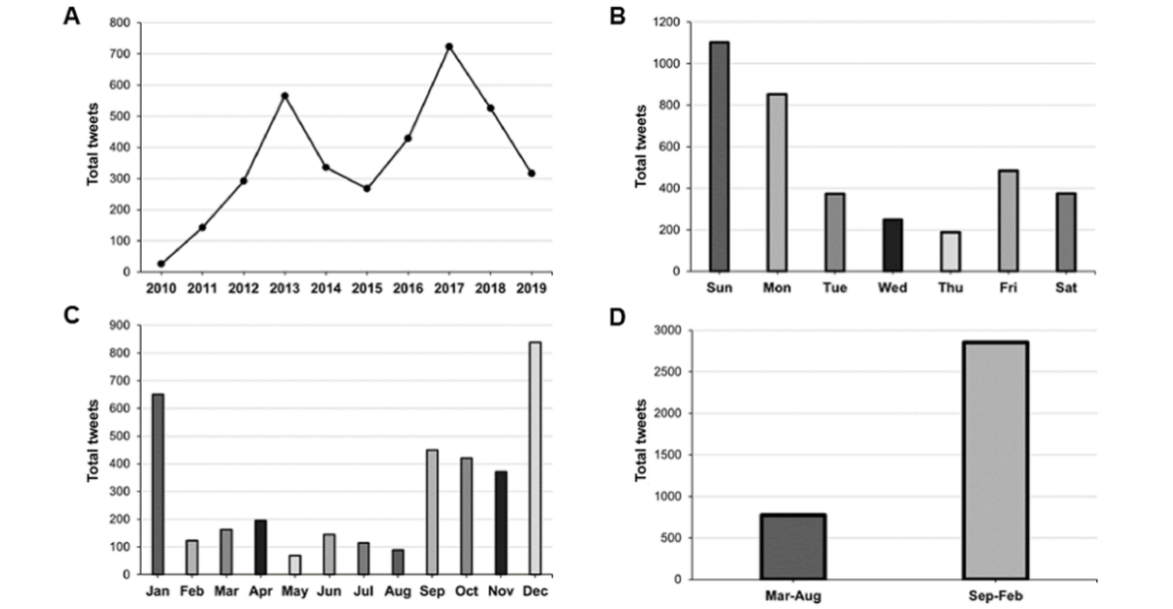
Trends in Twitter mentions of the fencing response. (A) The annual number of tweets that mention the fencing response increased overall from 2010 to 2019. (B) The highest number of tweets occurred on Sunday, followed by Monday. (C) The total number of tweets varied by month, with most occurring between September and January. (D) The years were divided into two 6-month periods roughly representing the American football season (September-February) and its offseason (March-August). There were more than 3 times as many tweets that mentioned the fencing response during the months of September-February compared to March-August.

The annual number of tweets mentioning the fencing response increased overall from 2010 to 2019, with notable peaks in 2013 and 2017 (Figure 1A). The highest number of tweets occurred on Sunday followed by Monday, and we observed a decrease as the week went on until Friday and Saturday (Figure 1B). The total number of tweets varied by month, with most tweets occurring in the latter half of the calendar year (Figure 1C). We chose to divide the years into two 6-month periods roughly representing the American football season (September to February) and its off-season (March to August). There were more than 3 times as many tweets mentioning the fencing response between September and February compared to the months from March to August (Figure 1D). A seasonal regression analysis further examined the relationship between the number of tweets and time (month of the year). The model was a significant predictor of the monthly number of tweets (*F*_1,12_=2.454, *P*=.007) and explained 22.1% of variation in the monthly number of tweets (*R*^2^=0.221). Month of the year had a significant effect on the number of tweets (*P*<.001) with December and January and having a significantly higher number of tweets than May (*P*=.002, *P*=.008, respectively).

**Figure 2:**
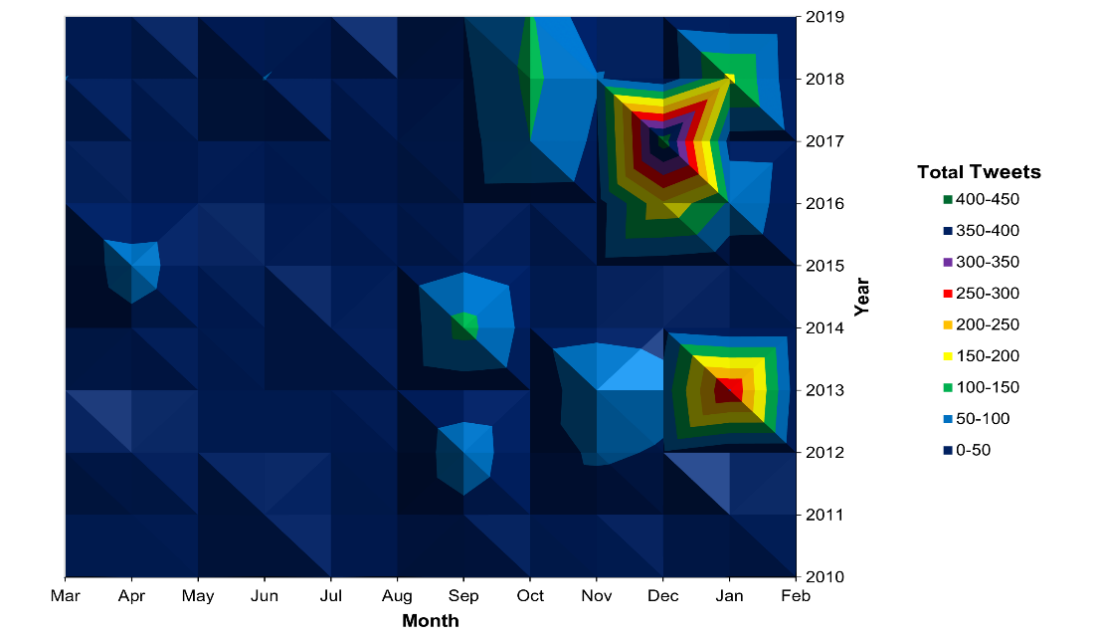
Tweets per month by year, 2010-2019. This is a topographical map of months by year, with the total number of tweets represented by elevation. In addition to the seasonal peak of tweets that mention the fencing response between September and February, there was an increase in the number of tweets over the years 2010-2019, with notable peaks in January 2013 and December 2017.

**Figure 3:**
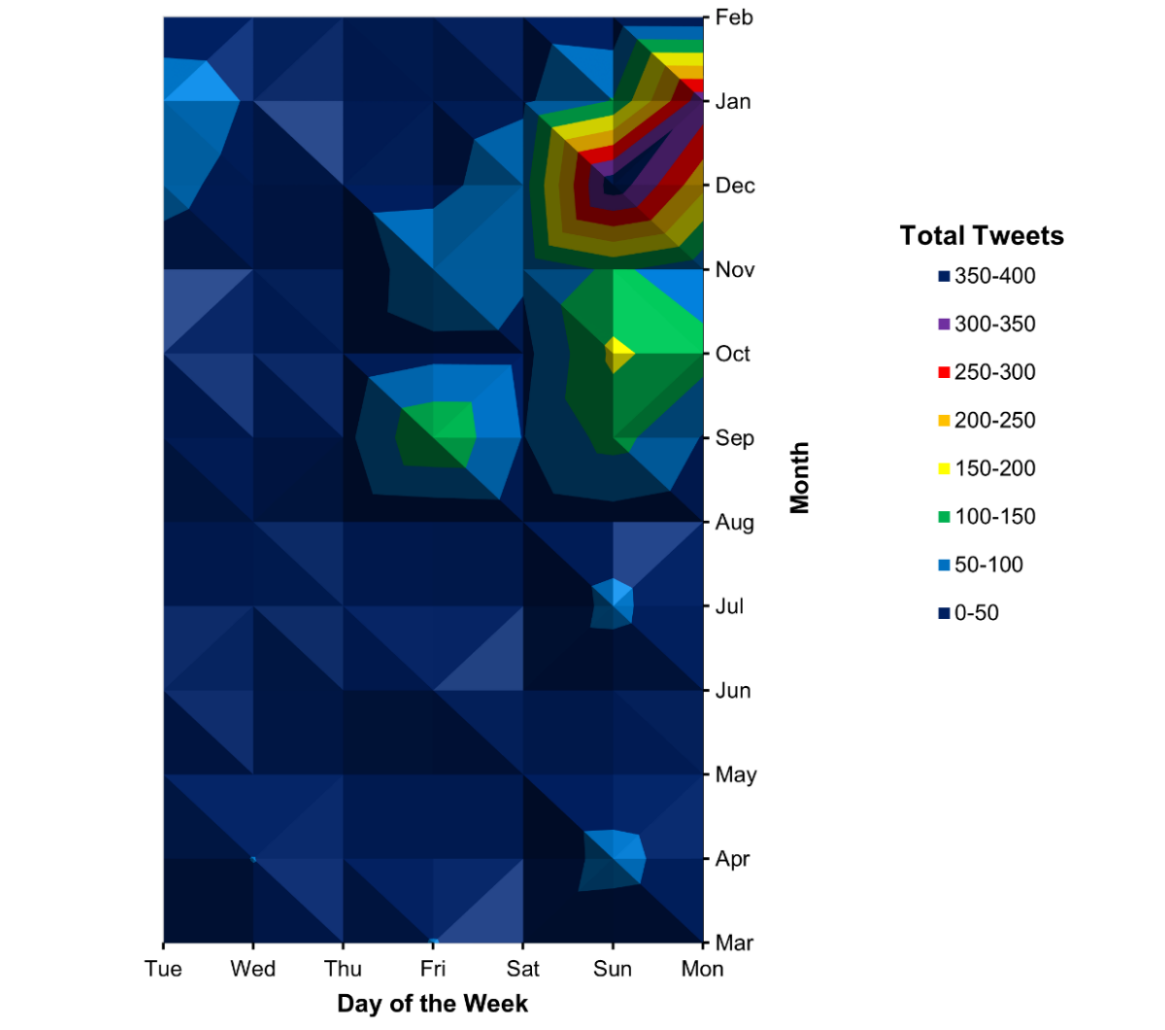
Tweets per day of the week by month. This topographical map shows that tweets that mention the fencing response were most common on Sunday and Monday during the months of September-February, which corresponds to the American football season.

In examining each year from 2010 to 2019 separately, a seasonal pattern was again observed, with most tweets occurring between September and February (Figure 2). During those months, tweets mostly occurred on Sundays and Mondays (Figure 3). This pattern was not observed during the months of March to August. Specific peaks in the number of Twitter mentions were reviewed to identify specific days with notable televised injury events that generated discussion on Twitter (Table 1).

**Table 1:** Notable events contributing to Twitter mentions and Wikipedia page views of the “fencing response”^a^.

Date	Event	Description	Number of tweets day prior to event	Number of tweets day of event and 3 days after	Number of Wikipedia page views day prior to event	Number of Wikipedia page views day of event and 3 days after
September 27, 2012	NFL^b^ game between Cleveland Browns and Baltimore Ravens	Browns wide receiver Josh Cribbs sustained a TBI^c^ on a punt return	0	25	83	1034
January 20, 2013	NFL game between New England Patriots and Baltimore Ravens	Patriots running back Stevan Ridley had a TBI with loss of consciousness. An article was subsequently published on the website *Bleacher Report* entitled, “Stevan Ridley's Concussion: Biomechanics of His Injury, 'Fencing Response'”	0	284	3527	19,485
November 10, 2013	English Premier League soccer match between Manchester United and Arsenal	Manchester United defender Nemanja Vidic sustained a TBI in a collision with goalkeeper David de Gea and was taken to the hospital	2	27	91	400
November 28, 2013	NFL game between Pittsburgh Steelers and Baltimore Ravens	Steelers running back Le’Veon Bell sustained a TBI during a collision with an opposing player	0	26	99	1881
April 26, 2015	English Premier League soccer match between Chelsea and Arsenal	Chelsea midfielder Oscar dos Santos Emboaba Júnior was taken to the hospital after sustaining a TBI in a collision with opposing goalkeeper David Ospina	0	65	101	17,806
January 9, 2016	NFL game between Pittsburgh Steelers and Cincinnati Bengals	Steelers wide receiver Antonio Brown sustained a TBI after being hit by an opposing player	0	40	166	20,363
January 16, 2016	NFL game between Kansas City Chiefs and New England Patriots	Chiefs defensive back Jamell Fleming sustained a TBI on a punt return after a hit by an opposing player	16^d^	27	414	8128
December 3, 2016	National Collegiate Athletic Association football game between the University of Oklahoma and Oklahoma State University	Over the course of the game, 3 University of Oklahoma players—Dede Westbrook, Jordan Parker, and Samaje Perine—were removed from play due to apparent TBIs	1	60	177	1275
October 15, 2017	NFL game between Washington Commanders and San Francisco 49ers	Commanders safety Montae Nicholson made helmet-to-helmet contact with 49ers receiver Pierre Garcon. The hit knocked Nicholson’s helmet off. He was evaluated for TBI and returned to play after about 20 minutes, despite displaying apparently involuntary arm extension and leg twitching immediately following the hit	0	54	239	3534
December 10, 2017	NFL game between Houston Texans and San Francisco 49ers	Texans quarterback Tom Savage sustained an apparent TBI after being tackled by an opposing player. He returned to play following a brief evaluation by medical staff. However, he was later removed from the game after a second evaluation revealed signs of a head injury	1	392	206	21,886
January 7, 2018	NFL game between Buffalo Bills and Jacksonville Jaguars	Bills quarterback Tyrod Taylor sustained a TBI when his head struck the ground as he was tackled by an opposing player. Taylor laid on the field for several minutes while being evaluated and was ultimately removed from the game	2	36	351	7742
June 15, 2018	Soccer World Cup match between Morocco and Iran	Morocco’s Noureddine Amrabat was removed from play and taken to the hospital after sustaining a TBI in a collision with an opposing player	0	47	341	6337
October 6, 2018	NFL game between Pittsburgh Steelers and Baltimore Ravens^e^	Steelers quarterback Mason Rudolph sustained a TBI after being hit by multiple Ravens defenders. He was removed from play and taken to the hospital	0	94	133	4905
October 7, 2018	NFL game between Los Angeles Rams and Seattle Seahawks^e^	Rams wide receiver Brandin Cooks sustained a TBI after a helmet-to-helmet collision with an opposing player	0	94	148	4934

^a^The table lists several notable examples of injury events that prompted discussion of the fencing response on Twitter and Wikipedia page views. The list is not meant to be exhaustive but serves to explain several of the spikes in Twitter mentions over the years 2010-2019.

^b^NFL: National Football League.

^c^TBI: Traumatic brain injury.

^d^Tweets that mentioned the fencing response prior to the event on January 16, 2016 were all referencing the prior notable event from January 9, 2016.

^e^Tweets and the number of Wikipedia page views prior to and on October 7, 2018 include references to both the notable event on October 6, 2018, and the notable event on October 7, 2018.

### Page Views of the Fencing Response Wikipedia Article Increased Over Time and Have Patterns of Seasonality

**Figure 4:**
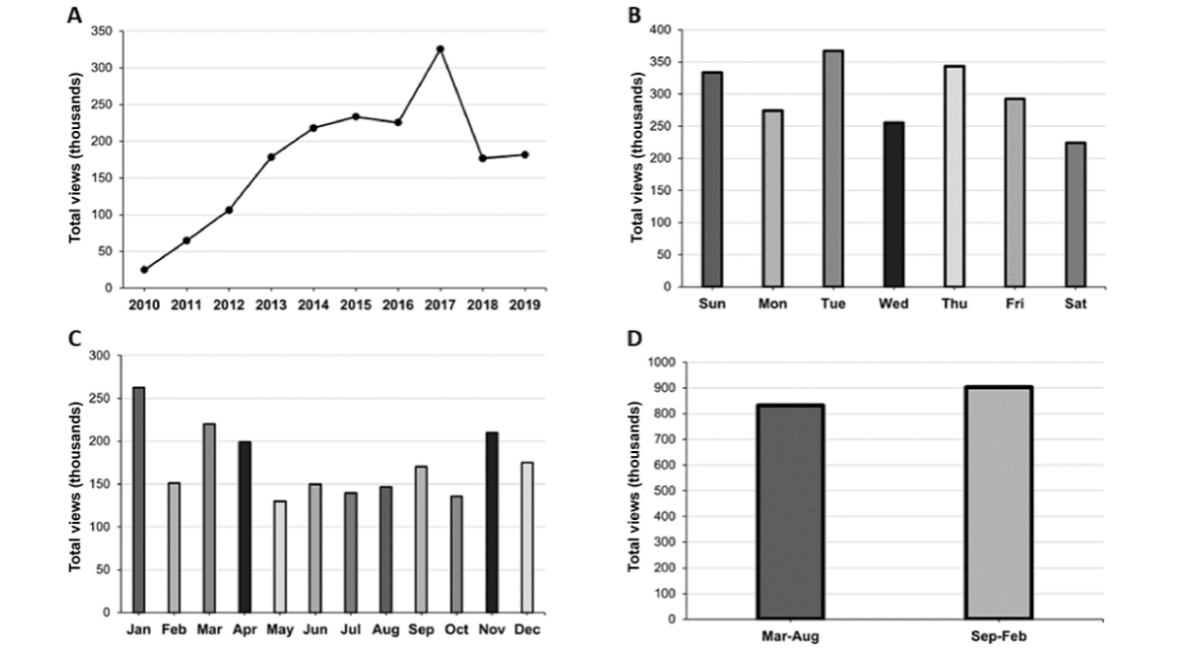
Trends in page views of the “Fencing Response” Wikipedia article. (A) The annual number of page views increased overall from 2010 to 2019. (B) Page views by day of the week varied with no clear pattern (eg, weekend vs weekday). (C) Monthly page views varied with January representing the peak month. (D) Dividing the years in half revealed only slight seasonal variation, with more page views occurring in September-February.

**Figure 5:**
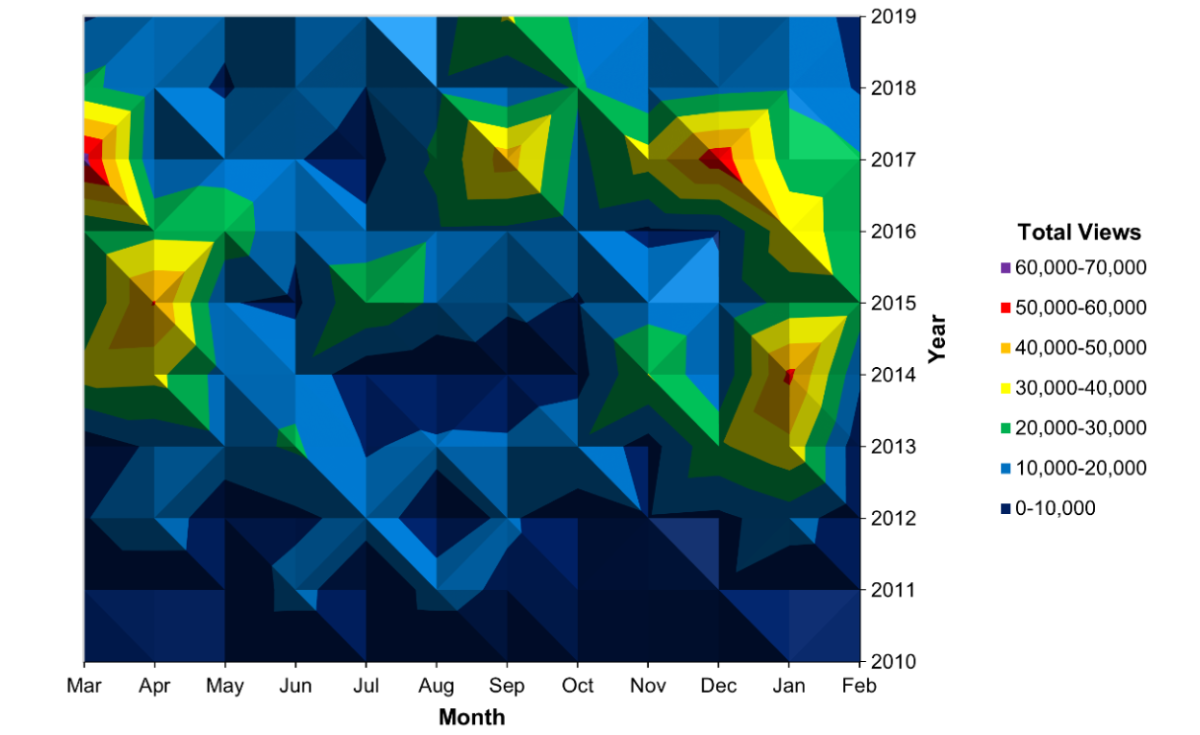
Page views of the “Fencing Response” Wikipedia article per month by year, 2010-2019. Article page views increased overall from 2010 to 2019. The topographical map does not appear to demonstrate a clear seasonal pattern; rather, it identifies multiple peaks across various months and years.

The annual number of page views of the *Fencing Response* Wikipedia article increased overall from 2010 to 2019 (Figure 4A). Page views by day of the week varied with no clear pattern (eg, weekend vs weekday; Figure 4B), and monthly page views varied with January representing the peak month (Figure 4C). We observed slight seasonal variation, with more page views occurring between September and February (Figure 4D). A seasonal regression analysis further examined the relationship between Wikipedia fencing response page views and time (month of the year). The model was a significant predictor of the monthly number of page views (*F*_1,12_=3.57, *P*<.001) and explained 29.2% of variation in the monthly number of page views (*R*^2^=0.292). Month of the year had a significant effect on the number of Wikipedia page views (*P*<.001) with January having significantly higher page views than May (*P*=.009). While article page views increased overall from 2010 to 2019, there were multiple peaks across combinations of months and years (Figure 5). Individual peaks were linked with notable injury occurrences in televised professional sports (Table 1).

## Discussion

Research published in peer-reviewed journals serves as the foundation of society’s scientific knowledge, and still the content remains out of reach to the public. Publicly available platforms, like Twitter and Wikipedia, vary in quality and have the ability to reach a broad audience. The 2009 peer-reviewed article on the fencing response published in the journal *Medicine & Science in Sports & Exercise* has been viewed approximately 320 times in the 5 years prior to the writing of this paper [14]. The *Fencing Response* Wikipedia article, on the other hand, has been viewed over 1 million times over the same period. Therefore, if an individual is aware of the fencing response, especially outside of the brain injury field, in all likelihood they learned about it through a publicly available platform rather than the original scientific paper.

Twitter mentions of the fencing response and page views of the *Fencing* *Response* Wikipedia article increased overall from 2010 to 2019, following the publication of the original peer-reviewed article that described the fencing response. Twitter mentions peaked during the American football season (September to February), especially on Sundays and Mondays and December and January overall had a higher number of Twitter mentions than May. This suggests that more discussion of the fencing response occurred on and following the National Football League (NFL) gameday. Wikipedia article page views did not appear to have peaks corresponding to specific days of the week, and we did not observe differences in the number of page views when dividing the year in half (September to February vs March to August). However, we observed seasonality across months, where the number of page views in January was significantly higher than the number of page views in May. Furthermore, the number of Wikipedia page views was orders of magnitude greater than the number of tweets.

Specific peaks in Twitter mentions were identified in January 2013 and December 2017. These and other minor peaks were associated with notable events that generated discussion on social media and also led to increased page views on the Wikipedia article (Table 1). For example, the January 2013 peak was associated with an NFL game between the New England Patriots and Baltimore Ravens on January 20, 2013, in which Patriots running back Stevan Ridley had a head injury with loss of consciousness. An article was subsequently published on the popular sports website *Bleacher Report* entitled, “Stevan Ridley's concussion: biomechanics of his injury, 'fencing response’” [31]. The day prior to the televised injury, there were zero tweets that mentioned the fencing response. On the day of the injury and 3 days following the injury, there were 284 tweets that mentioned the fencing response (Table 1). Wikipedia page views increased approximately 5.5 times on the 3 days following the injury (Table 1).

The December 2017 peak was associated with an NFL game between the Houston Texans and San Francisco 49ers on December 10, 2017, in which Texans quarterback Tom Savage sustained an apparent TBI after being tackled by a 49ers defender. Savage returned to play following a brief evaluation by medical staff. However, he was later removed from the game after a second evaluation revealed signs of a TBI. The day prior to the injury, there was 1 tweet that mentioned the fencing response. Whereas, on the day of the injury and 3 days following the injury, there were 392 tweets that mentioned the fencing response (Table 1). Additionally, Wikipedia page views increased approximately 106 times on the 3 days following the injury (Table 1). These examples demonstrate more discussion and self-education on the fencing response, and head injury, on and following American football game days.

Of note, the NFL added the fencing response (tonic posturing) as a sign of potential concussion to their Concussion Diagnosis and Management Protocol in 2017 [32]. American football is the most popular spectator sport in the United States, and recognition of the fencing response as a “red-flag” sign of TBI by the NFL could further increase awareness among large audiences [33]. As role models in the medical management of athletes, the actions of the NFL can influence decisions in collegiate, high school, club, and recreational sports.

### Limitations

One limitation of the Twitter data in this report is the restriction to primary tweets and retweets, without analysis of “likes.” Further analyses, including visualization of a Twitter conversation with Treeverse, may more completely characterize the Twitter conversation by hierarchical indexing of primary tweets, retweets, and “liked” tweets. However, some of these analyses require an index tweet to drive the conversation, whereas most discourse on the fencing response originates from observed experience, not a single tweet. It should also be noted that Twitter and Wikipedia are available worldwide to anyone with internet access. Thus, the patterns reported here may be explained by differing usage rates of these platforms in countries across the globe and by the popularity of American football. The United States has, by far, more Twitter users than any other country at approximately 77 million. Following are Japan and India with approximately 59 million and 24 million users, respectively [34]. Twitter’s user base in other countries is comparatively small. Wikipedia, on the other hand, while still more popular in the United States than any other country, is used more broadly [35]. This could explain why Twitter mentions of the fencing response appear to occur mostly during the American football season, while *Fencing Response* Wikipedia page views do not demonstrate the same seasonal pattern. Additionally, we limited our data collection to Twitter and Wikipedia for the accessibility of APIs to extract data in the public domain; other platforms exist to demonstrate and examine patterns of interest such as Google Trends, YouTube, Instagram, Facebook, and LinkedIn.

### Conclusions

In this study, data from Twitter and Wikipedia were used to demonstrate an overall increase in public discussion and awareness of the fencing response as an indicator of TBI over time. The results show weekly and seasonal patterns of increased discussion that align roughly with the American football season. Peaks in Twitter mentions and Wikipedia page views represented national-level events that created public discourse about the fencing response. Public platforms such as Twitter and Wikipedia can play an effective role in science communication and greater awareness of the fencing response among coaches, athletic trainers, and sports organizations has the potential to help with “return-to-play” and other medical care decisions after a potential TBI.

## Abbreviations

API: application programming interfaces
ED: Emergency department
NFL: National Football League
TBI: traumatic brain injury

## Data Availability

Data analyzed during the current study are available from the corresponding author upon request.

## References

[1] Taylor CA, Bell JM, Breiding MJ, Xu L (2017). Traumatic brain injury-related emergency department visits, hospitalizations, and deaths: United States, 2007 and 2013. MMWR Surveill Summ.

[2] National Center for Injury Prevention and Control, Division of Unintentional Injury Prevention, Centers for Disease Control and Prevention (2015). Report to Congress: traumatic brain injury in the United States: epidemiology and rehabilitation. Centers for Disease Control and Prevention.

[3] Zaloshnja E, Miller T, Langlois JA, Selassie AW (2008). Prevalence of long-term disability from traumatic brain injury in the civilian population of the United States, 2005. J Head Trauma Rehabil.

[4] Ma VY, Chan L, Carruthers KJ (2014). Incidence, prevalence, costs, and impact on disability of common conditions requiring rehabilitation in the United States: stroke, spinal cord injury, traumatic brain injury, multiple sclerosis, osteoarthritis, rheumatoid arthritis, limb loss, and back pain. Arch Phys Med Rehabil.

[5] Maas AIR, Menon DK, Adelson PD, Andelic N, Bell MJ, Belli A, Bragge P, Brazinova A, Büki András, Chesnut RM, Citerio G, Coburn M, Cooper DJ, Crowder AT, Czeiter E, Czosnyka M, Diaz-Arrastia R, Dreier JP, Duhaime AC, Ercole A, van Essen TA, Feigin VL, Gao G, Giacino J, Gonzalez-Lara LE, Gruen RL, Gupta D, Hartings JA, Hill S, Jiang JY, Ketharanathan N, Kompanje EJO, Lanyon L, Laureys S, Lecky F, Levin H, Lingsma HF, Maegele M, Majdan M, Manley G, Marsteller J, Mascia L, McFadyen C, Mondello S, Newcombe V, Palotie A, Parizel PM, Peul W, Piercy J, Polinder S, Puybasset L, Rasmussen TE, Rossaint R, Smielewski P, Söderberg Jeannette, Stanworth SJ, Stein MB, von Steinbüchel Nicole, Stewart W, Steyerberg EW, Stocchetti N, Synnot A, Te Ao B, Tenovuo O, Theadom A, Tibboel D, Videtta W, Wang KKW, Williams WH, Wilson L, Yaffe K, InTBIR ParticipantsInvestigators (2017). Traumatic brain injury: integrated approaches to improve prevention, clinical care, and research. Lancet Neurol.

[6] Blennow K, Brody DL, Kochanek PM, Levin H, McKee A, Ribbers GM, Yaffe K, Zetterberg H (2016). Traumatic brain injuries. Nat Rev Dis Primers.

[7] Giordano KR, Lifshitz J, Honeybul S, Kolias AG (2021). Pathophysiology of traumatic brain injury. Traumatic Brain Injury: Science, Practice, Evidence and Ethics.

[8] Rabinowitz AR, Levin HS (2014). Cognitive sequelae of traumatic brain injury. Psychiatr Clin North Am.

[9] Silver JM, Kramer R, Greenwald S, Weissman M (2001). The association between head injuries and psychiatric disorders: findings from the New Haven NIMH Epidemiologic Catchment Area Study. Brain Inj.

[10] Fehr SD, Nelson LD, Scharer KR, Traudt EA, Veenstra JM, Tarima SS, Liu XC, Walter KD (2019). Risk factors for prolonged symptoms of mild traumatic brain injury: a pediatric sports concussion clinic cohort. Clin J Sport Med.

[11] Narayana S, Charles C, Collins K, Tsao JW, Stanfill AG, Baughman B (2019). Neuroimaging and neuropsychological studies in sports-related concussions in adolescents: current state and future directions. Front Neurol.

[12] Kim HJ, Tsao JW, Stanfill AG (2018). The current state of biomarkers of mild traumatic brain injury. JCI Insight.

[13] Patricios J, Fuller GW, Ellenbogen R, Herring S, Kutcher JS, Loosemore M, Makdissi M, McCrea M, Putukian M, Schneider KJ (2017). What are the critical elements of sideline screening that can be used to establish the diagnosis of concussion?: a systematic review. Br J Sports Med.

[14] Hosseini AH, Lifshitz J (2009). Brain injury forces of moderate magnitude elicit the fencing response. Med Sci Sports Exerc.

[15] Zimmerman KA, Cournoyer J, Lai H, Snider SB, Fischer D, Kemp S, Karton C, Hoshizaki TB, Ghajari M, Sharp DJ (2022). The biomechanical signature of loss of consciousness: computational modelling of elite athlete head injuries. Brain.

[16] Bruce SL, Dorney K (2020). Posturing responses in concussions sustained by elite American football players. Int J Athl Ther Train.

[17] Beitchman JA, Burg BA, Sabb DM, Hosseini AH, Lifshitz J (2022). The pentagram of concussion: an observational analysis that describes five overt indicators of head trauma. BMC Sports Sci Med Rehabil.

[18] Pfundner A, Schönberg T, Horn J, Boyce RD, Samwald M (2015). Utilizing the Wikidata system to improve the quality of medical content in Wikipedia in diverse languages: a pilot study. J Med Internet Res.

[19] Sun F, Yang F, Zheng S (2021). Evaluation of the liver disease information in Baidu Encyclopedia and Wikipedia: longitudinal study. J Med Internet Res.

[20] Weiner SS, Horbacewicz J, Rasberry L, Bensinger-Brody Y (2019). Improving the quality of consumer health information on wikipedia: case series. J Med Internet Res.

[21] Yacob M, Lotfi S, Tang S, Jetty P (2020). Wikipedia in vascular surgery medical education: comparative study. JMIR Med Educ.

[22] Scaffidi MA, Khan R, Wang C, Keren D, Tsui C, Garg A, Brar S, Valoo K, Bonert M, de Wolff JF, Heilman J, Grover SC (2017). Comparison of the impact of Wikipedia, UpToDate, and a digital textbook on short-term knowledge acquisition among medical students: randomized controlled trial of three web-based resources. JMIR Med Educ.

[23] Farič N, Potts HWW (2014). Motivations for contributing to health-related articles on Wikipedia: an interview study. J Med Internet Res.

[24] Taneja SL, Passi M, Bhattacharya S, Schueler SA, Gurram S, Koh C (2021). Social media and research publication activity during early stages of the COVID-19 pandemic: longitudinal trend analysis. J Med Internet Res.

[25] Hart M, Stetten N, Islam S, Pizarro K (2017). Twitter and public health (part 2): qualitative analysis of how individual health professionals outside organizations use microblogging to promote and disseminate health-related information. JMIR Public Health Surveill.

[26] Academic research access: advance your research objectives with public data on nearly any topic. Developer Platform: Twitter API.

[27] Barrie C, Ho JC (2021). AcademictwitteR: an R package to access the Twitter Academic Research Product Track v2 API endpoint. J Open Source Softw.

[28] Meissner P, R Core Team (2020). Public subject attention via Wikipedia page view statistics. Comprehensive R Archive Network.

[29] Keyes O, Lewis J (2020). An API client for Wikimedia traffic data. Comprehensive R Archive Network.

[30] Fencing response. Wikipedia.

[31] Siebert D (2013). Stevan Ridley's concussion: biomechanics of his Injury, 'fencing response'. Bleacher Report.

[32] Ellenbogen RG, Batjer H, Cardenas J, Berger M, Bailes J, Pieroth E, Heyer R, Theodore N, Hsu W, Nabel E, Maroon J, Cantu R, Barnes R, Collins J, Putukian M, Lonser R, Solomon G, Sills A (2018). National football league head, neck and spine committee's concussion diagnosis and management protocol: 2017-18 season. Br J Sports Med.

[33] Simpson-Wood T, Wood RH (2018). When popular culture and the NFL collide: fan responsibility in ending the concussion crisis. Marquette Sport Law Rev.

[34] (2022). Leading countries based on number of Twitter users as of January 2022 (in millions). Statista.

[35] (2018). Wikimedia Traffic Analysis Report: Wikipedia Page Views Per Country.

